# Breaking the cycle of malaria treatment failure

**DOI:** 10.3389/fepid.2022.1041896

**Published:** 2022-12-14

**Authors:** Maciej F. Boni

**Affiliations:** ^1^ Department of Biology, Center for Infectious Disease Dynamics, Pennsylvania State University, University Park, PA, United States; ^2^ Nuffield Department of Medicine, Centre for Tropical Medicine and Global Health, University of Oxford, Oxford, United Kingdom

**Keywords:** malaria, drug resistance, multiple first-line therapies, artemisinin combination therapies, artemisinin resistance

## Abstract

Treatment of symptomatic malaria became a routine component of the clinical and public health response to malaria after the second world war. However, all antimalarial drugs deployed against malaria eventually generated enough drug resistance that they had to be removed from use. Chloroquine, sulfadoxine-pyrimethamine, and mefloquine are well known examples of antimalarial drugs to which resistance did and still does ready evolve. Artemisinin-based combination therapies (ACTs) are currently facing the same challenge as artemisinin resistance is widespread in Southeast Asia and emerging in Africa. Here, I review some aspects of drug-resistance management in malaria that influence the strength of selective pressure on drug-resistant malaria parasites, as well as an approach we can take in the future to avoid repeating the common mistake of deploying a new drug and waiting for drug resistance and treatment failure to arrive. A desirable goal of drug-resistance management is to reduce selection pressure without reducing the overall percentage of patients that are treated. This can be achieved by distributing multiple first-line therapies (MFT) simultaneously in the population for the treatment of uncomplicated falciparum malaria, thereby keeping treatment levels high but the overall selection pressure exerted by each individual therapy low. I review the primary reasons that make MFT a preferred resistance management option in many malaria-endemic settings, and I describe two exceptions where caution and additional analyses may be warranted before deploying MFT. MFT has shown to be feasible in practice in many endemic settings. The continual improvement and increased coverage of genomic surveillance in malaria may allow countries to implement custom MFT strategies based on their current drug-resistance profiles.

## Introduction

In malaria-endemic countries, antimalarial drug policy has always been guided by identifying the most effective therapy—often by age group, clinical severity, or pregnancy status—and recommending it as the first-line treatment option (1). Chloroquine (CQ) was the most effective, safest, and least expensive drug for decades. Despite the emergence of chloroquine resistance in the late 1950s and 1960s and its subsequent spread in the following decades, CQ was recommended as a first-line antimalarial into the 1990s and continued to be sold into the early part of this century (2–4) far beyond its useful period as an effective clinical intervention (5). Sulfadoxine-pyrimethamine (SP) became, by default, the best available broadly-applicable therapy in the late 1990s when CQ cure rates in Africa fell below 50% (6). Then, with the rapid spread of SP resistance (7), the success of clinical trials of artemisinin-based drugs in the 1990s (8–12), and the development of artemisinin-based combination therapies (ACTs) shortly thereafter (13, 14), the World Health Organization (WHO) recommended in 2005 that ACTs be adopted globally as first-line therapy against uncomplicated falciparum malaria (15). ACTs have been used globally for more than 15 years and have likely made an enormous contribution to the decline of malaria that has been seen during this time (16, 17). Hidden in this triumph is the knowledge that the honeymoon with artemisinin-based drugs will eventually be over. Early signs of drug resistance, first observed in Cambodia from 2006 to 2008 (18, 19) and most recently in Africa (20, 21), have led us to a predictable reenactment of the scenarios that played out with chloroquine, SP, mefloquine, and the many other antimalarials whose efficacy was eroded by the emergence and spread of drug resistance.

Antimalarial chemotherapy is one of the two most important components of malaria control as it works both to cure patients and reduce onward transmission, lowering both prevalence and incidence. For many decades, we have been refortifying our chemotherapeutic defenses, when first-line drugs stopped working, by slowly replacing the therapies we use to treat malaria—always reacting to the problem of drug resistance rather than anticipating it. Great fortunes and energies have been spent gathering the data for public health response, and almost always too late. This despite the fact that the eventual outcome of resistance evolution is predictable, in some cases certain, given that we broadly expose the parasite population to the same strong selection pressure when we roll out a newly chosen drug as a replacement for a failing therapy. As public health planners, why are we content to react to the emergence of drug-resistant parasites rather than acting early and acting preemptively? It is imperative that we break out of this cycle of failure and replacement.

A key positive enabler of early action against drug resistance is the global genomic surveillance framework that has been built over the past 15 years (22–24). With genotyped *Plasmodium falciparum* collections routinely reaching thousands of samples and molecular marker validation standards (25, 26) reaching a point where molecular marker counts alone (20, 21) are now sufficient to make public health assessments of drug resistance, we are in a position where drug-resistant genotype frequencies can be identified and reported at the 1% level or lower. This is critical because early detection of drug resistance allows for early interventions to be planned (27).

In addition, the development of multiple types of ACTs over the past 15 years—a total of six currently pre-qualified by WHO (28)—has presented us with a possible solution. In deciding which therapy we should recommend when multiple therapies are available, equally safe, and equally efficacious, the best answer may be that a deliberate recommendation should be made to deploy all therapies simultaneously, with different patients receiving different treatments. The rationale behind such a strategy is that it would delay drug-resistance evolution. In the same way that combination therapy presents a more complex survival problem for blood-stage malaria parasites, the deployment of multiple first-line therapies (MFT) forces parasites to adapt to simultaneous multiple lethal challenges (29). If the parasite has mastered drug X in patient A, it will see drug Y in patient B next month. The major difference between combination therapy and MFT is that combination therapy offers more simultaneity in antimalarial drug action, as parasites see multiple drugs during the same 48-h replication cycle. Under MFT, parasites in symptomatic hosts will wait several weeks before seeing another drug—an acceptable compromise as this approach still makes evolutionary adaptation difficult for the parasites.

Planning a public health strategy around drug-resistance prevention allows us to anticipate drug-resistance evolution rather than react to it. Persuading National Malaria Control Programs (NMCP) to reformulate their guidelines to allow for multiple types of therapies to be used—with custom approaches appropriate to every region's (1) supply chain constraints, (2) operational capacity, (3) current prevalence level, (4) current knowledge on circulating resistance markers, and (5) current drug recommendations for chemoprophylaxis—will build a level of flexibility into malaria control plans that will allow for a better feedback loop between malaria surveillance and malaria control activities. This would allow for MFT or MFT-like strategies to be trialed, evaluated, and modified, allowing each NMCP to pursue a fit-for-purpose strategy with the best long-term chances of substantial reductions in malaria prevalence and antimalarial drug resistance.

## Evidence and rationale for MFT

**Figure 1:**
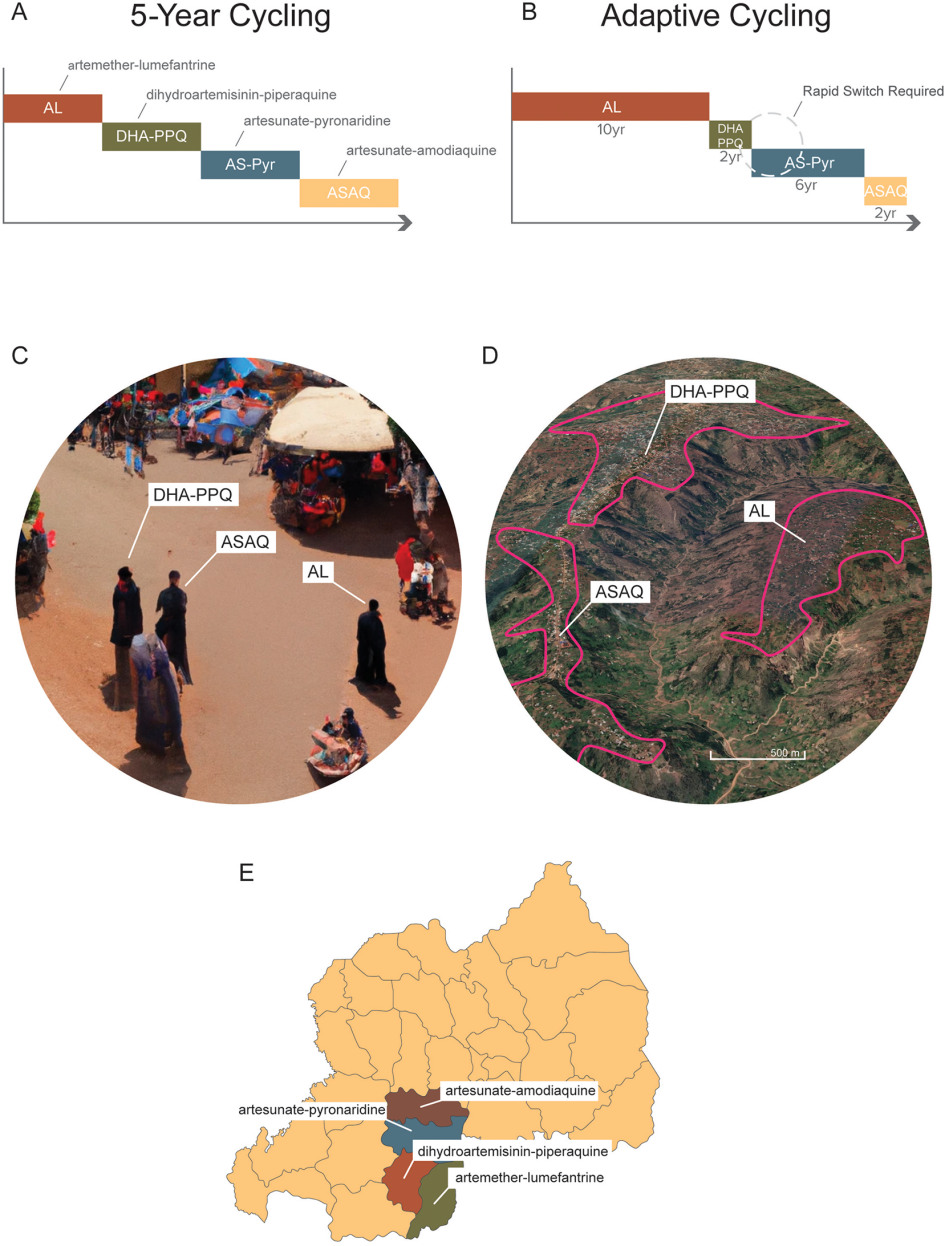
Different long-term deployment strategies of when multiple ACTs are available. (**A**) A 5-year cycling strategy where each ACT is pre-scheduled to be used for 5 years exactly; after each 5-year period the national first-line recommendation is switched to a different ACT. (**B**) An adaptive cycling strategy, currently recommended by WHO, where ACTs (or other therapies) are replaced when 10% treatment failure is surpassed. This means that over long periods, different therapies will be used for different amounts of time. It also means that switches from an old therapy to a new one will typically occur with a delay, sometimes with a substantial delay if surveillance is delayed. (**C**) Multiple first-line therapies (MFT) deployed with random allocations of therapies to clinics, pharmacies, and other health facilities. In this scenario, three individuals in the same community could be simultaneously treated with three different ACTs. (**D**) MFT deployed by village or health post or health facility. In this scenario, a central point of contact in the health system—e.g., a village health worker, or a health facility director—would be responsible for ensuring that all malaria cases in their catchment area were treated with one ACT, chosen at a higher administrative level. (**E**) MFT deployed at the district or province level and coordinated nationally. In this scenario, distribution of different ACTs to different administrative regions would be controlled centrally by the National Malaria Control Program.

The preferred method for comparing long-term population-level treatment strategies aimed at minimizing the detrimental effects of drug resistance is *in silico* experimentation using mathematical models of pathogen transmission and evolution. The rationale for this approach is that population-level field trials are expensive and impractical for an outcome that may not occur for a decade or more. Mathematical modeling results analyzing drug resistance evolution in bacteria and malaria suggest that the simultaneous population-level use of drugs is better than rotating those same drugs (30–36). Comparisons in these modeling analyses are usually made between simultaneous deployment and cycling, as these are the two most feasible options for long-term drug stewardship when multiple therapies are available (Figure 1). Drug cycling is evaluated in two common schemes, either rotating drugs in and out on a fixed predetermined schedule (e.g., every 5 years) or replacing drugs only when treatment failure rates become too high [for malaria, this is done at the WHO-recommended level of 10% treatment failure (28)]. A third option sometimes included in these comparisons is a hypothetical combination therapy of all available drugs (31, 32). This option is typically not available in practice as a newly proposed combination needs to undergo extensive safety and efficacy testing, while rotation and MFT strategies can be deployed immediately. Nevertheless, modeling results do indicate that combination therapies dramatically reduce the likelihood of long-term resistance evolution with a small additional risk of driving multi-drug resistance (37).

The reason that drug-resistance management strategies work at all is that they take advantage of drug-resistant pathogens’ reduced Darwinian fitness, or their fitness cost (38–41). The fitness cost associated with drug resistance means that in the absence of drug pressure, drug-resistant genotypes should be outcompeted by drug-sensitive genotypes, both within patients and in the population at large. In fact, this is the only major leverage that we have against drug resistance of any kind: with no fitness cost of resistance, resistant genotypes would simply continue spreading after emergence even under low levels of drug coverage (42, 43). Given that drug-resistance mutations are typically associated with a fitness cost [there are exceptions to this rule (44)], the evolutionary game for any pathogen encountering drug pressure is that it has to keep the cost of carrying drug resistance genes (*c_R_*) below the cost of reduced survival in the presence of drugs (*c_D_*). The policy counter-move for a control program would be to keep treatment coverage *f* below the ratio *c_R_*/*c_D_* in order to avoid driving drug resistance too strongly (45), but this comes with the major disadvantage that some individuals will not receive treatment. The challenge then becomes how to keep the use of individual drugs low but still treat as many patients as possible. The best solution appears to be to distribute many different types of drug simultaneously, keeping the population coverage of each drug relatively low, but keeping overall treatment levels high.

There are three key reasons why a strategy of simultaneous drug distribution like MFT is associated with better long-term population-level health outcomes than the various forms of drug cycling.

First, MFT creates more pharmacodynamic heterogeneity for the parasite population, delaying the emergence of resistance and slowing down resistance evolution if resistant genotypes have already emerged. Essentially, the parasites see a more diverse drug environment under an MFT policy. If a single parasite acquires a beneficial drug-resistance mutation today, 1 month later it may be replicating in a patient who is being treated with a different therapy to which the parasite has no resistance mutations; cycling policies do not enjoy this benefit. As an example, if three drugs are being deployed simultaneously, there is only a one in three chance that a parasite's newly acquired drug-resistance mutation will confer any survival advantage in the short term. We are essentially constructing a complex set of detours in the parasites’ fitness landscape making it nearly impossible for them to climb fitness peaks. If drugs are being cycled out and replaced, the parasites see the same drug for one entire phase of the cycle, which could last as long 5 or 10 years, and the parasite's evolutionary problem is made easy—evolve resistance to the currently used drug. Evolution occurs much more readily in a constant environment than in a rapidly changing environment (46).

Second, cycling strategies have a particular disadvantage in the way that they rapidly generate drug resistance during each cycling phase. To illustrate, consider the replacement of chloroquine by SP in the late 1990s. Sulfadoxine-pyrimethamine was adopted widely in Africa as a response to high failure rates for CQ treatment. Mutations in the *dhfr* and *dhps* genes that confer SP resistance (47) were already present or emerged soon thereafter (depending on location), and these new resistant genotypes began to spread (7). What we did not notice at the time is that the previous era of CQ-resistance evolution had made evolution easier for SP-resistant genotypes. Instead of having to outcompete wild-type drug-sensitive malaria parasites, the new SP-resistant genotypes were placed into a competition with CQ-resistant genotypes that were established at high frequencies in the late 1990s. We know today that chloroquine resistance carries a substantial fitness cost (40, 48). Thus, SP-resistant genotypes were handed an easy evolutionary challenge and won quickly.

This is a general problem with cycling strategies, that eventually we lose the ability to use the parasites’ fitness cost against them. Once a large amount of drug resistance is established—as inevitably occurs each time we notice that surveillance has been insufficient and that drug resistance is widespread—resistance evolution is made easier for the parasites regardless of the new drug that will be chosen to replace the currently failing therapy. After the failing drug is replaced, newly emerging resistant genotypes are placed in a competition with other resistant parasites, not the optimally fit wild-types. Cycling policies, by generating large amounts of one kind of drug resistance, make it easier for other resistant types to invade and spread. An argument can be made that there is not enough evidence to estimate magnitude of this effect, and whether it is meaningful in most treatment contexts, but field conditions will never be appropriate to perform a controlled study on this specific hypothesis. Caution should push us to understand the evolutionary theory behind this mechanism and to recommend further *in silico* analyses assessing its risk and magnitude.

Third, cycling strategies can cause epidemiological rebounds. The classic shape of a resistance epidemic is that prevalence drops when a successful treatment is introduced, it begins to rise when resistance emerges, and then levels off to a new equilibrium when drug-resistance establishes itself as the major phenotype in the population (33). But for each specific epidemiological scenario the dynamics of prevalence changes may not be so well behaved, and soft landings (49–52) to theoretically-predicted equilibria are not guaranteed. In reality, when drug-resistance emerges, an epidemic wave may surge upwards overshooting its expected equilibrium prevalence [see figure 1 in Nguyen et al. (35)]. The prevalence level will settle back to equilibrium eventually, but the public health damage at this point will have already been done. The mechanisms behind “epidemiological swings” like these can be explained with an analogy from physics (upon whose foundations the theory of epidemiological dynamics is built). Imagine a pendulum at rest hanging from a tripod—it is at equilibrium. If you move the tripod to another part of the room, the pendulum will settle to a new equilibrium, but not before swinging back and forth for a period of time. The faster you move, the more violent the swings. This is exactly what happens when an epidemiological system is jolted from one equilibrium to another—in this case, an endemic equilibrium prior to a drug switch and a new endemic equilibrium after a drug switch. Under this scenario, epidemiological swings are likely to occur, and we have no standardized or evidence-based approaches available to control them. Evidence for epidemiological rebounds and epidemics overshooting their expected dynamics have been documented for influenza (50), malaria (53), and SARS-CoV-2 (54, 55).

As all of these mechanisms are supported by scientifically mature theory in evolutionary epidemiology, they form part of the evidence base on which to make sound decisions in malaria policy. *In silico* and *in vitro* approaches may be used to test the robustness and limits of these hypotheses, but prospective field studies are impractical for this purpose as running a trial is no different than implementing a policy. An important robustness consideration for modeled ACT deployments, cycling and MFT approaches alike, is that all ACTs contain an artemisinin component necessitating separate evaluations of artemisinin-resistance evolution and partner-drug resistance evolution (56).

Two arguments against MFT need to be addressed when considering its deployment. First, the use of several drugs simultaneously in a population may allow different resistant genotypes to be brought together, through recombination, into a single multi-drug resistant genotype (1, 57). For the strongest version of this effect, we would need genetic recombination to occur at high frequency in high-transmission regions where (1) mosquitoes would be likely to bite individuals with multiple clonal parasite populations which could then recombine, and (2) multiply-feeding mosquitoes would be likely to have both bites occurring on infected individuals, allowing the two malaria clones from these individuals to recombine in the mosquito. However, even in these scenarios, recombination between two different drug-resistant genotypes may still be rare as drug-resistant genotype frequencies typically remain at low levels during the emergence phase of drug-resistance evolution; recombination events between two rare types would, in theory, be doubly rare. A simulation study focused on this exact question—on emergence patterns of double and triple resistance—showed that these multi-drug resistant (MDR) genotypes generally emerged later under MFT than under cycling policies, and that the total MDR risk (total number of MDR frequency-days, summed over five different MDR genotypes) was between 22% and 90% lower under MFT than under cycling policies (36).

Second, all therapies do not have the same efficacy and all drug-resistant genotypes are not equally resistant. If one therapy has higher efficacy than all others, MFT deployment means that a portion of patients will not be treated with the highest efficacy treatment. If one therapy generates drug resistance with a very high treatment failure rate, this may lead to sub-optimal health outcomes if this therapy is deployed at all. For this reason, simulation studies evaluating optimal drug policy need to be specifically parameterized with therapeutic efficacy estimates on both wild-type parasites and resistant genotypes (58). When efficacy estimates differ greatly between candidate therapies (a subjective evaluation must be made here), this is a sign that any MFT deployment needs to be custom evaluated for a particular health system and geography. As an example, AL use drives the evolution of double-resistant genotypes with efficacies approaching 70% (58), but the double-resistant genotype to DHA-PPQ drops treatment efficacy to 42% (59). A modeling study focused on AL and DHA-PPQ deployment in Burkina Faso showed that MFT is not optimal when these two therapies are available due to the predicted early and rapid rise in piperaquine resistance (60).

## MFT in practice

If avoiding the detrimental effects of repeated drug replacement is to become a mainstay of population-level malaria policy, at both national and international levels, it is important to determine how this type of simultaneous drug deployment can be achieved in practice.

In Ghana, the use of multiple first-line therapies was added into the 2009 national anti-malaria drug policy, as a straightforward recommendation that multiple types of ACTs should be purchased and distributed, explicitly noting the benefits of delaying and slowing drug resistance (61). The guidelines listed ASAQ as first-line therapy and AL and DHA-PPQ as alternative first-line therapies for patients that cannot tolerate ASAQ. However, no specific provision was made for distribution of different ACTs. In Indonesia, the 2011 national malaria guidelines listed the first-line antimalarial as “ACT, including dihydroartemisinin-piperaquine, artesunate-amodiaquine,” and the government provided both for free to health centers and clinics around the country, although the trend eventually shifted to DHA-PPQ because of its perceived higher efficacy. The Indonesian guidelines explicitly stated that “malaria case management is an integral part of malaria control programmes and should be based on a clear understanding of epidemiology … as well as data on the pattern of parasite resistance to antimalarial drugs” (62). These types of statements in national guidelines are an appropriate starting point for communicating to national and local health authorities the benefits of long-term resistance management and multiple first-line therapies.

Since 2005, some African countries have moved to adopt, in practice, multiple ACTs as first-line. Angola began distributing AL and ASAQ to health facilities in May 2006 and in March 2012 DHA-PPQ was added as a third first-line option (63, 64). Choice of prescription is left to individual health facilities and physicians. Interruptions in the supply chain, inconsistent stocking, and inadequate funding persist in Angola's drug procurement but it is not clear that these result from the choice to deploy multiple therapies at once (65). Burkina Faso adopted AL and ASAQ simultaneously in 2005 (66), and recent field trials and interviews have indicated a general level of acceptance of MFT as a worthwhile national drug policy (67–69). The major challenge in Burkina Faso was patient and provider preference for AL, which led to AL being used as treatment of choice for about 70% of uncomplicated falciparum cases by 2018 (70). According to the 2021 World Malaria Report, nine African countries, Brazil, Costa Rica, Thailand, India, and China recommend two or more therapies as first-line for confirmed uncomplicated falciparum malaria (16).

Changes to national guidelines are important steps in pushing forward the adoption of more complex malaria control policies. However, additional challenges will have to be overcome at the levels of suppliers, health facilities, providers, and patients. Some logistical challenges were known from the earliest MFT discussions, including (1) addressing perceptions of inequality among the different therapies, (2) improving inventory management, (3) ensuring adherence to equal distribution levels of the different therapies used, and (4) understanding the health-system incentives that currently work against the establishment of MFT (71, 72). Comparing the absolute costs of drug procurement and additional inventory holdings versus the DALY reductions one would expect from MFT due to lower malaria burden and fewer treatment failures, economic and epidemiological modeling indicates that using more drugs is better than using fewer drugs (34). However, this cost comparison needs to be tested in practice. This means that evaluations of supply chains, distribution chains, drug costs, stocking costs, and health staff retraining will have to be ground-truthed with field evaluations that measure all the additional activity created for local health providers due to MFT implementation (69).

Recent studies suggest that perceptions of MFT are changing and moving towards acceptance (67, 69). The simplest starting point for communication around MFT is one that establishes the equivalence of certain antimalarial therapies (67, 71)—for example that AL, ASAQ, and AS-pyronaridine have equal efficacies and equal resistance risks—as this will help steer the health system to a uniform drug distribution in which each of these ACTs is prescribed or sold in approximately equal amounts. If the therapies are substantially different in efficacy or resistance risk, they should not be deployed together in a simple equal-distribution MFT policy. No distribution chain will be able to deliver a precise target distribution of antimalarial therapies, but the deployment of multiple therapies does not have to be perfectly uniform to achieve the resistance-delaying benefits afforded by MFT [figure 6 in Boni et al. (33), figure S24 in Nguyen et al. (35)]. The key question to answer is whether purchasing, distribution, and stocking can be made somewhat flexible at national and local levels; if this is possible, there will be multiple points in the health system where prescription numbers of the available therapies can be increased or decreased. Further work will be necessary to reduce prices of certain drugs and to measure the counts of different therapies that reach patients versus the counts that are put into the distribution system.

Recommendations have been made in the past that multiple therapies could be distributed in the form of adult/pediatric formulations or by separating drugs into public sector and private sector sales (71). These particular distribution strategies have not yet been evaluated for feasibility, but a major limitation with these approaches is that they would allow for a maximum of two drugs to be distributed simultaneously. A more complex age-based scheme can be created to distribute more than two therapies, but questions will follow whether this is simpler than distributing therapies in a purely random age-independent manner. An additional evaluation (by simulation) will be needed to determine whether an age-based distribution would have any unexpected epidemiological outcomes as the relationship among age, symptoms, and infection varies greatly among different malaria endemicity settings.

## Role of genomic surveillance in drug-resistance management

The evolution and fixation of drug resistance occurs gradually then suddenly (73). Drug-resistant genotypes can circulate at frequencies of 0.0001 or 0.001 for many years before they are eventually noticed by surveillance systems at frequencies (recently observed in Rwanda and Uganda) ranging from 0.02 to 0.22 (20, 21, 74). This period of complacency, prior to molecular confirmation of circulating drug resistance (75), is currently unavoidable as detecting low-frequency genotypes requires sample sizes in the thousands to be submitted for sequencing each year. If molecular markers for new resistant phenotypes are not yet available, the challenge is even greater as resistance must be identified *via* an increase in treatment failures in a therapeutic efficacy study (TES). This is one of the current challenges with resistance markers to amodiaquine and lumefantrine—certain loci in the *pfmdr1* and *pfcrt* genes are known to be associated with resistance to these two partner drugs (76–84) but a summary across studies shows that these effect sizes are likely small (58).

The next key step in allowing genomic surveillance to improve drug-resistance management approaches is the introduction of feasible and cost-effective combinations of good genomic surveillance habits and frequent TESs. The cost component of genomic surveillance directly trades off with sample size, which directly influences the surveillance system's statistical power to detect a low-frequency genotype circulating at an early stage. A major cost in both molecular surveys and TESs is the time the surveillance system has to wait before results are available (85). A health economic analysis on the annual sample size of sequenced falciparum parasites and the annual number of patients enrolled in TES studies should yield an optimum for both numbers that would minimize future cases and deaths by enabling early control of drug resistance, but these studies have not yet been done. The current prevailing opinion is that TESs are not done frequently enough, that molecular surveillance needs to be more geographically comprehensive, and that both need to make results available in real time (27, 85).

Early identification of drug resistance, in all malaria contexts, will allow appropriate responses to be put into place. Speed is one of the key elements of a successful response, as the lack of a response ensures that drug resistance evolution continues in a singular direction. Critically, early detection allows for consideration of more types of response options. Early detection of treatment failure or resistant genotypes may allow an MFT option to be put into place that would reduce selection pressure on both the currently circulating resistant genotype and future genotypes that would be selected for by other drugs. Late detection constrains the decision-making process, as the current therapy then needs to be completely removed and replaced, restarting the pattern of cyclic drug replacements that in the past has led to strong selection and high levels of drug failure. Breaking this cycle of failure and replacement may be the key in allowing national malaria control programs to transition to a new paradigm of long-term management of low-level circulation of drug-resistant malaria genotypes.

## Data Availability

The original contributions presented in the study are included in the article/Supplementary Material, further inquiries can be directed to the corresponding author.
